# Predictors of Obesity among Gut Microbiota Biomarkers in African American Men with and without Diabetes

**DOI:** 10.3390/microorganisms7090320

**Published:** 2019-09-05

**Authors:** Elena Barengolts, Stefan J. Green, George E. Chlipala, Brian T. Layden, Yuval Eisenberg, Medha Priyadarshini, Lara R. Dugas

**Affiliations:** 1 Division of Endocrinology, Diabetes, and Metabolism, Department of Medicine, University of Illinois at Chicago, Chicago, IL 60612, USA; 2 Section of Endocrinology, Department of Medicine, Jesse Brown VA Medical Center, Chicago, IL 60612, USA; 3 Sequencing Core, Research Resources Center, University of Illinois, Chicago, IL 60612, USA; 4 Research Informatics Core, Research Resources Center, University of Illinois, Chicago, IL 60612, USA; 5 Department of Public Health Sciences, Parkinson School of Health Sciences and Public Health, Loyola University Chicago, Maywood, IL 60153, USA

**Keywords:** gut microbiota, BMI, body mass index, CD14, cluster of differentiation 14 protein, EndoCab, endotoxin core antibody, LBP, lipopolysaccharide-binding protein, SCFA, short-chain fatty acids, zonulin, butyric, propionic, obesity, type 2 diabetes mellitus, African American men, cortisol

## Abstract

Gut microbiota and their biomarkers may be associated with obesity. This study evaluated associations of body mass index (BMI) with circulating microbiota biomarkers in African American men (AAM) (*n* = 75). The main outcomes included fecal microbial community structure (16S rRNA), gut permeability biomarkers (ELISA), and short-chain fatty acids (SCFAs, metabolome analysis). These outcomes were compared between obese and non-obese men, after adjusting for age. The results showed that lipopolysaccharide-binding protein (LBP), the ratio of LBP to CD14 (LBP/CD14), and SCFAs (propionic, butyric, isovaleric) were higher in obese (*n* = 41, age 58 years, BMI 36 kg/m^2^) versus non-obese (*n* = 34, age 55 years, BMI 26 kg/m^2^) men. BMI correlated positively with LBP, LBP/CD14 (*p* < 0.05 for both) and SCFAs (propionic, butyric, isovaleric, *p* < 0.01 for all). In the regression analysis, LBP, LBP/CD14, propionic and butyric acids were independent determinants of BMI. The study showed for the first time that selected microbiota biomarkers (LBP, LBP/CD14, propionic and butyric acids) together with several other relevant risks explained 39%–47% of BMI variability, emphasizing that factors other than microbiota-related biomarkers could be important. Further research is needed to provide clinical and mechanistic insight into microbiota biomarkers and their utility for diagnostic and therapeutic purposes.

## 1. Introduction

Obesity is a common problem worldwide. The worldwide prevalence of obesity nearly tripled between 1975 and 2016 [1]. In 2016, more than 1.9 billion adults, 18 years and older, have been classified as overweight, and of these, over 650 million have been described as obese. Overweight and obesity are defined as abnormal or excessive fat accumulation that can impair health. Body mass index (BMI) is a simple index of weight-for-height that is commonly used to classify overweight and obesity. It is expressed as a person’s weight in kilograms divided by the square of his height in meters (kg/m^2^). For adults, the World Health Organization (WHO) defines overweight and obesity as a BMI ≥25 and ≥30, respectively. BMI provides the most useful population-level measure of overweight and obesity [1]. Increased BMI is a major risk factor for type 2 diabetes (T2D) and more than 90% of type 2 diabetics are overweight or obese [1,2]. In 2016, an estimated 1.6 million deaths have been attributed to diabetes and commonly, death occurs before the age of 70 years. The WHO estimates that T2D was the seventh leading cause of death in 2016 [2]. There are multiple emerging treatments for obesity and T2D, but the management of both conditions remains challenging to physicians and burdensome to society [3,4,5,6].

The role of gut microbiota and related biomarkers and their contributions to obesity and T2D is a subject of ongoing research. The gut microbiota may contribute to obesity by generating excess energy from non-digestible nutrients and by increasing absorption of high-density nutrients [7,8]. In humans, the large bowel anaerobic Gram-negative bacteria ferment previously indigestible complex carbohydrates, converting them into short-chain fatty acids (SCFAs) [9,10]. The main SCFAs include acetic (C2:0), propionic (C3:0), and butyric (C4:0), which account for about 95% of the biologically significant SCFAs produced in the colon [9,10,11]. The SCFAs are primarily absorbed through the portal vein [12], providing about 10% of the total host energy requirement [7,8]. The role of SCFAs in obesity is unclear. On the one hand, there is evidence to suggest that fecal SCFAs are higher in obese compared to lean individuals [13,14,15,16], implying a role for SCFAs in obesity development. Conversely, SCFAs can induce gut hormones (e.g., GLP-1 and PYY) [17,18] and can be associated with decrease in appetite, weight loss and glucose control [18,19,20].

Similarly, biomarkers for gut microbiota metabolites and byproducts may increase gut permeability and nutrient absorption and, therefore, additionally contribute to obesity [7,8]. The main byproduct responsible for inducing increased gut permeability and microbiota translocation into host interior is lipopolysaccharide (LPS, endotoxin). LPS-related serological surrogate markers of gut permeability and bacterial translocation include LPS-binding protein (LPB), LPS core antibody (EndoCab), soluble CD14 (CD14), and zonulin. LBP is a soluble acute-phase protein, EndoCab is a measure of LPS burden (it is impracticable to measure LPS directly), CD14 is a cluster of differentiation 14 protein from a family of pattern-recognition receptors [21]. Zonulin (haptoglobin 2 precursor) is a protein that modulates the permeability of tight junctions between cells in the wall of the digestive tract [22]. All these biomarkers play important and divergent role in microbiome’s influence on host physiology and pathogenesis of obesity [23,24,25,26,27,28,29].

While the gut microbiota have been associated with the development of obesity [7,8] and T2D [28,30], there is increasing evidence that they are also associated with other chronic conditions including hypertension [31], cardiovascular disease [32], and even some psychiatric disorders [33]. To date, however, there is a dearth of studies exploring associations between obesity and gut microbiota-related biomarkers in African American men (AAM). The focus of this exploratory study was to investigate associations between gut microbiota-related biomarkers and obesity in AAM. To accomplish this, we explored determinants of body mass index (BMI) among microbiota-related biomarkers in a cohort consisting of 41 obese and 34 non-obese AAM. The gut microbiota-related biomarkers included SCFAs (acetic, propionic, butyric, and isovaleric) and permeability/translocation markers (LBP, EndoCab, CD14, and zonulin).

## 2. Material and Methods

### 2.1. Design and Participants

**Figure 1:**
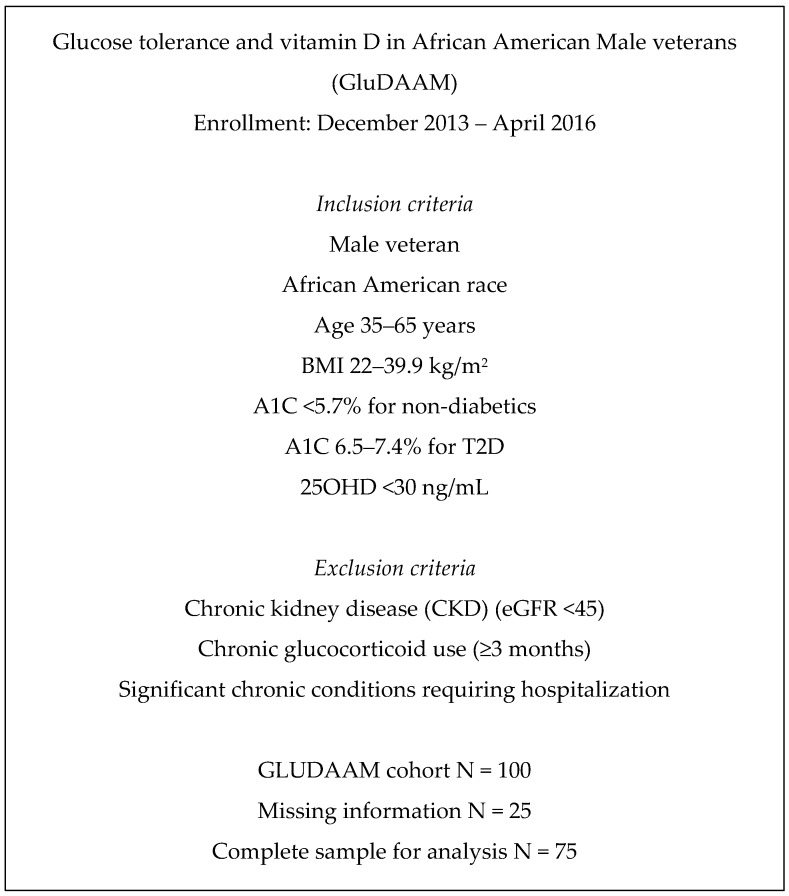
Overall Design of Study.

The study participants were African American men (AAM) presenting in ambulatory clinics at an urban Veteran Health Administration Medical Center, as part of the Glucose tolerance and vitamin D in African American Male veterans (GluDAAM) study (Figure 1). GluDAAM is a cross-sectional study assessing metabolic characteristics in African American men (AAM) [34]. The inclusion criteria were glycohemoglobin A1c (HbA1c) < 5.7% without T2D or 6.5%–7.4% with T2D, age 35–70 years, BMI 22–39.9 kg/m^2^, and 25OH-vitamin D (25OHD) < 30 ng/mL. Exclusion criteria were chronic kidney disease (stages 3b, 4, and 5), chronic glucocorticoid use (3 months or longer), taking non-metformin antihyperglycemics, and presence or history of significant health conditions requiring recent (within 6 months) hospitalization. After providing written informed consent, participants completed a questionnaire that included demographic, lifestyle, and health information. In a single-visit study, personnel captured biometrics (height and weight), and specimens were collected for hormonal and biomarker analysis. Past medical history was confirmed by review of electronic medical records. Lifestyle factors included current smoking (“yes” or “no”) and exercise (“yes” for 2 h or more of exercising per week). A short questionnaire assessed overall dietary habits by a number of servings per week. Foods including fruits, vegetables, nuts, porridge, breakfast cereal, milk, and tea were categorized as “healthy”, whereas servings of potato, fried and canned food, pasta, and soft drinks were categorized as “unhealthy”. The biometric measurements and calculations were performed as previously [34,35], and included body weight, height and body mass index (BMI, kg/m^2^). The prevalence of chronic conditions, e.g., hypertension, cardiovascular disease, cancer, etc. was assessed by the Charlson index of chronic disease [34,35]. Psychiatric disorders (“yes” or “no”) were included separately because these are not included in the Charlson index and due to the high prevalence of these disorders in the cohort. The measured hormonal levels included testosterone, representing the essential male hormone, and cortisol, representing the stress-related hormone. The Jesse Brown VA Medical Center Institutional Review Board approved the study. The recruitment dates were from 1 December 2013 to 15 April 2016.

### 2.2. Biomarker Analysis

Analysis of SCFAs, leaky-gut biomarkers, and gut microbiota were explored to evaluate associations with obesity. Specifically, hormonal assays were performed in a clinical laboratory and a core research laboratory, applying laboratory standards of care and references as previously described [27,34,35]. Hormonal assays for cortisol and testosterone were performed in a clinical laboratory. Blood was collected and serum stored at −80 °C until assayed. 

### 2.3. Serum Short Chain Fatty Acids

Serum was collected in a fasting state and stored at −80 °C until analyzed. Serum acetic, butyric, propionic and isovaleric acids were measured by liquid chromatography with tandem mass spectrometry (LC-MS/MS) after derivatization using Shimadzu protocol, where solutions, mixing, and standards were used per protocol [36]. The LC-MS/MS system used was an Agilent 1290 UPLC coupled to a QTRAP 6500 mass spectrometer (AB Sciex, Concord, ON, Canada) equipped with an ESI source and operated in the negative-ion mode. Chromatographic separations were performed on a Waters BEH C18 (2.1 × 100 mm, 1.7 mm) UPLC column, using water:formic acid (100:0.01, *v*/*v*; solvent A) and acetonitrile:formic acid (100:0.01, *v*/*v*; solvent B) as the mobile phase for gradient elution. The column flow rate was 0.35 mL/min; the column temperature was 40 °C, and the autosampler was kept at 4 °C. The binary solvent elution gradient was optimized at 15% B for 2 min, 15%–55% B in 9 min, and then held at 100% B for 1 min. The column was equilibrated for 3 min at 15% B between injections. The measurements were performed at the Mass Spectrometry Core facility of the University of Illinois at Chicago (UIC) Research Resource Center (RRC).

### 2.4. Serum Translocation Biomarkers

Serum leaky-gut biomarkers included soluble CD14 (CD14), lipopolysaccharide (LPS)-binding protein (LBP), LPS core antibody (EndoCab), and zonulin. Commercial enzyme-linked immunosorbent sandwich assays (ELISA) kits were used as follows: CD14 (DC140; Quantikine, R&D Systems, Minneapolis, MN, USA), LBP (HK315-02; Hycult Biotechnology, Uden, The Netherlands), EndoCab (EndoCAb IgA-HK504-IGG; Hycult Biotechnology, Uden, The Netherlands), and zonulin (MBS706368; MyBioSource, Inc., San Diego, USA). Optical density was measured at 450 nm (ELX808 BioTek) and concentrations were determined using assay standard curves.

### 2.5. Microbial Community Evaluation

The fecal microbial community structure was characterized using amplicon-based microbiome profiling as described previously [34,37]. Stool samples were collected in plastic tubes and stored at −80 °C until extraction. Genomic DNA was extracted and processed for microbial community analysis using a two-stage PCR protocol [38] followed by high-throughput sequencing as described previously [27,34]. Briefly, the widely used primer, targeting the V3-V4 variable regions of the 16S rRNA gene of Bacteria, was used, and sequencing was performed on an Illumina MiSeq sequencer using standard V3 chemistry with paired-end, 300 base reads. Fluidigm sequencing primers, targeting the CS1 and CS2 linker regions (so called “common sequences”), were used to initiate sequencing. Library preparation and sequencing was performed at the UIC Sequencing Core (UICSQC). 

### 2.6. Basic Sequence Processing

The basic sequence processing was performed as described previously [27,34]. Briefly, forward and reverse reads were merged using the software package PEAR [39]. Primer sequences were identified using Smith–Watermann alignment. Reads that lacked either of the primer sequences were discarded. Sequences were then trimmed based on quality scores using a modified Mott algorithm with PHRED quality threshold of *p* = 0.01. After trimming, any sequences less than 275 bp were discarded. Chimeric sequences were identified using the USEARCH61 algorithm with the GreenGenes 13_8 reference sequences. QIIME v1.8 was used to generate OTU tables and taxonomic summaries. The resulting sequence files were merged with sample information. Operational taxonomic unit (OTU) clusters were generated in a de novo manner using the UCLUST algorithm with a 97% similarity threshold. Taxonomic annotations for each OTU were determined using the UCLUST algorithm and GreenGenes 13_8 reference with a minimum similarity threshold of 90%. Taxonomic and OTU abundance data were merged into a single OTU table. Prior to any analyses, the OTU table was filtered to remove any sequences from mitochondria or chloroplasts and then rarified to a depth of 5600 counts per sample. The filtered and rarified OTU table was then used to generate summaries of absolute abundances of taxa for all phyla, classes, orders, families, genera, and species [27,34].

### 2.7. Statistical Analysis

Metabolic indicators: Statistical analysis was performed as described previously [27,34]. The groups were specified a priori; however, as this was an exploratory study, we did not perform a sample size calculation a priori. This analysis was dedicated to an exploratory outcome, evaluating microbiota-related biomarkers’ contribution to obesity. Therefore, the groups were stratified based on BMI as non-obese (BMI < 30 kg/m^2^) and obese (BMI ≥ 30 kg/m^2^). Data were described as mean ± standard deviation (SD) for continuous variables or number (percent) for categorical variables. For categorical variables, data were number (%) for “yes” answer, and the chi-square and logistic regression were used to denote statistical significance. For a continuous variables t-test, Pearson correlation, and linear regression, adjusting for age, were used as appropriate. For the multivariate regressions, BMI was a dependent variable. An alpha *p*-value of <0.05 was considered statistically significant. All metabolic analyses were performed in STATA v.14 (College Station, TX, USA). 

Microbiota analyses: For each sample, raw sequence counts from the rarefied dataset were used for analysis as previously described [27,34]. The values for the taxa were reported as the total sequence counts. Prior to group testing and correlation analyses, all taxonomic summaries were filtered to remove taxa with a relative abundance of below 1% across the total dataset. Shannon indices (alpha diversity) were calculated on the rarefied genus data in the R programming environment using the vegan library [40]. The Kruskal–Wallis one-way analysis of variance and Mann–Whitney tests were used to compare Shannon indices between groups, as described previously [27,34]. Correlations were tested between marker levels determined via biochemical and hormonal assays and all taxonomic units using a Kendall Tau (τ) test of correlation. The resulting *p*-values were adjusted for multiple testing using the false-discovery rate (FDR) correction of Benjamini and Hochberg as described previously [34]. All statistical analyses were performed using R 3.2.3 statistical software. 

Data access. The amplicon sequence data from this study have been submitted to the NCBI Sequence Read Archive (http://www.ncbi.nlm.nih.gov/Traces/sra/sra.cgi) under the BioProject PRJNA389481.

## 3. Results 

### 3.1. General Characteristics

The general characteristics showed many expected differences between non-obese and obese groups (Table 1). Obese participants (*n* = 41) were slightly older than non-obese (*n* = 34) (57.9 ± 4.0 vs. 55.3 ± 5.0 years, *p* = 0.05). The obesity-related characteristics (body weight and BMI) were higher in obese than non-obese (*p* < 0.01 for both) as expected. Specifically, in non-obese vs. obese, body weight was 79.5 ± 8.2 vs. 110.4 ± 11.4 kg (*p* < 0.01), while BMI was 25.6 ± 2.4 vs. 35.5 ± 2.5 kg/m^2^ (*p* < 0.01). Lifestyle assessment showed that self-reported smoking and exercising were similar in both groups while non-obese reported eating about two more servings of healthy food per week compared to obese (12.45 ± 3.50 vs. 10.10 ± 3.24 servings/wk, *p* < 0.01). More obese participants exercised for ≥ 2 h/wk (56%) compared to non-obese participants (44%); however, the difference did not reach statistical significance (*p* = 0.46). The prevalence of diabetes was greater in obese participants (non-obese vs. obese: 1 ± 3% vs. 37 ± 90%, *p* < 0.01), as expected; however, psychiatric disorders were lower in obese (21 ± 51%) than non-obese (27 ± 79%, *p* < 0.05), and overall disease burden measured by the Charlson index was similar (non-obese vs. obese: 1.62 ± 0.98% vs. 2.15 ± 0.97%, *p* = 0.13). Obese compared to non-obese participants had higher HbA1c (6.70 ± 0.28% vs. 5.22 ± 0.29%, *p* < 0.01) and lower testosterone (258.8 ± 105.1 vs. 401.7 ± 98.6 ng/dL, *p* < 0.01), as expected, while circulating 25OHD was similar (14.86 ± 4.26 vs. 17.12 ± 4.41 ng/mL, *p* = 0.10). Blood cortisol was slightly higher in the non-obese than the obese group (11.07 ± 3.32 vs. 9.06 ± 3.28 μg/dL, *p* = 0.02).

### 3.2. SCFAs and Translocation Biomarkers

Next, we examined the age-adjusted data for SCFAs and leaky-gut indices (Table 1). The comparisons showed that SCFAs, including propionic, butyric, and isovaleric, were higher in obese than non-obese (*p* < 0.01 for all), while no difference was seen for acetic acid. The leaky-gut biomarkers comparisons showed that the LBP-to-CD14 ratio (LBP/CD14) was higher (*p* = 0.01) and LBP was marginally higher (*p* = 0.05) in obese than non-obese participants. The other leaky-gut biomarkers (CD14, EndoCab, zonulin) were not different between the groups (Table 1). 

### 3.3. Determinants of Body Mass Index (BMI)

Because age tended to be lower among the non-obese participants, we adjusted all of our analyses for age, adjustments for HbA1c were not performed because of high collinearity between BMI and HbA1c (age-adjusted r = 0.78, *p* < 0.001). Correlation analysis (Table 2) showed that BMI was positively associated with SCFAs (propionic, butyric, isovaleric, *p* < 0.01 for all), as well as with LBP (*p* = 0.04) and LBP/CD14 (*p* < 0.01). Conversely, BMI negatively correlated with cortisol, testosterone, and healthy food servings (*p* < 0.01 for all) (Table 2). Next, we performed univariate and multivariate regression analyses. The independent parameters with a *p* < 0.1 in the univariate analysis were included in the multivariate analysis. Multivariate regression modeling included either butyric or propionic or isovaleric acid among other independent parameters since SCFAs were known to be metabolically related and highly collinear. Similarly, multivariate regression modeling included either LBP/CD14 or LBP among independent parameters since LBP and LBP/CD14 are also highly collinear. Multiple regression analysis showed that butyric and propionic acids, as well as LBP and LBP/CD14, were independent predictors of BMI (Table 3). The first model (Model-1), including butyric acid, LBP/CD14, and in addition age, testosterone, cortisol, healthy food servings, and psychiatric disorders, explained 47% of BMI variability (adjusted R^2^ = 0.47) (Table 3). The second model (Model-2), which included propionic instead of butyric acid and all independent parameters of Model-1 explained 42% of the variability in BMI (adjusted R^2^ = 0.42) (Table 3). Further modeling substituted LBP/CD14 with LBP (Table 3). Model-3 included LBP (not LBP/CD14) and all parameters of Model-1; this model explained 44% of the variability in BMI. Model-4 included LBP and all parameters of Model-2; this model explained 39% of BMI variability. Finally, neither isovaleric nor acetic acid or any other leaky-gut measures (EndoCab, CD14, and zonulin) were statistically associated with BMI. In all regression models, cortisol but not testosterone was negatively associated with BMI and was significant BMI determinant.

### 3.4. Microbial Community

**Figure 2:**
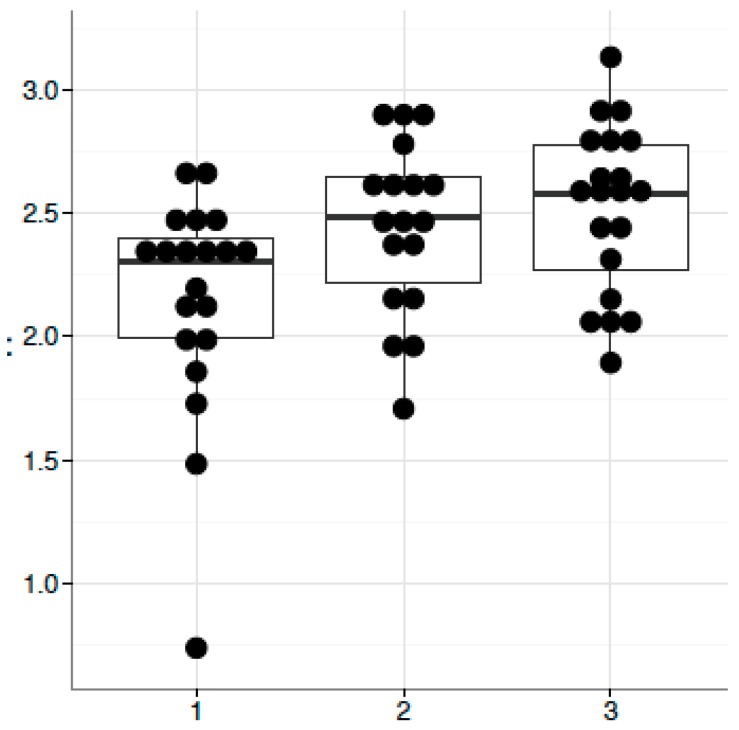
Association of cortisol with the Shannon index of microbiota diversity. Data are: *Y*-axis is Shannon index, *X*-axis: 1, 2, 3 are Tertiles (T) of cortisol level, *p* = 0.006 for T1 versus T3 by Mann–Whitney test.

BMI per se was not significantly associated with any identified microbiota species (*q* > 0.05), though there was a suggestive marginal trend for a negative association between *Catenibacterium* and BMI (*p* = 0.09). We then performed analysis of associations between microbiota and microbiota-related biomarkers (Table 4), as well as hormones (cortisol and testosterone) (Table 5). Among biomarkers, some associations were suggested by *p* < 0.05. However, none proved to be significant using FDR-adjusted *q* < 0.05, likely due to small number of participants for such analysis. The suggested associations (*p* < 0.05) included propionic acid and *Lachnospira* (*p* = 0.01, *q* = 0.18), CD14 and Enterobacteriaceae (*p* = 0.01, *q* = 0.18), LBP and Enterobacteriaceae (*p* = 0.03, *q* = 0.58), zonulin and *Catenibacterium* (*p* = 0.02, *q* = 0.46) (Table 4). Among hormones, cortisol—and not testosterone—showed mostly weak associations with low correlation coefficients. We also compared microbiota between the highest and lowest tertiles of cortisol and testosterone and observed significant difference in relative abundance of *Catenibacterium* for cortisol (*p* < 0.01, *q* < 0.05) (Table 5) but not testosterone tertiles. In addition, Shannon index analysis of microbiota diversity was significant only between the highest and lowest tertiles of cortisol (Figure 2).

## 4. Discussion

We explored the relationship between the gut microbiota, their metabolites and obesity in AAM. The main finding was that gut microbiota biomarkers were independent predictors of BMI and tended to be associated with the Enterobacteriaceae and Lachnospiraceae bacterial families. Specifically, LBP and LBP/CD14, as well as SCFAs (propionic and butyric), were independent predictors of BMI. Notably, for the first time, we showed that in AAM, SCFAs and intestinal permeability biomarkers were determinants of BMI and, together with age, diet, psychiatric disorders, and serum cortisol and testosterone, explained 39%–47% of BMI variability. To our knowledge, there are no published data that include all of these biomarkers for assessing their contribution to obesity. 

### 4.1. BMI Association with Microbiota Translocation Biomarkers

The microbiota translocation biomarker LBP/sCD14 was chosen for regression modeling since it had the highest association with BMI (adjusted *r* = 0.31, *p* < 0.01) among translocation biomarkers. The data from the current study for LBP/CD14 as an indicator of obesity agreed with previously published results [26,29]. For example, weight gain induced by overfeeding of healthy men resulted in an increased LBP/CD14 ratio, but not in LBP or CD14 concentrations per se, suggesting that the initial phase of weight gain was linked to the relative variations of LBP and CD14 [26]. Similarly, in obese premenopausal women, the use of yogurt in a randomized trial resulted in lower LBP/CD14 while LPS, LBP, CD14, and zonulin were not affected [29]. These changes occurred despite approximately 1.0 kg body weight gain in these women [29]. The only other translocation biomarker acting as a predictor of BMI in the current study was LBP. In previous publications, circulating LBP was frequently positively associated with obesity [25,41,42,43], although this was not found in all studies [44]. LBP was also associated with obesity-related disorders, including metabolic syndrome [41], insulin resistance [23], T2D [41], atherosclerosis [45], non-alcoholic fatty liver disease (NAFLD) [46], and hypogonadism [47]. In another prospective cohort study of 1312 men and women age 50–70 years, LBP was associated with BMI at baseline and at 6-year follow up, as well as with an increased risk of developing metabolic syndrome [48]. Correspondingly, weight loss after bariatric surgery was associated with lower LBP compared to pre-surgery values [42]. Therefore, the results of the present study correspond to the previously published data, although the role of LBP as a predictor of BMI was shown for the first time.

Mechanisms linking LBP to obesity appear to involve LBP production and activity in adipose tissue [49]. For example, adipose tissue LBP mRNA and LBP protein levels were longitudinally increased with weight gain and excessive fat accretion in both humans and mice and decreased with weight loss [49]. The LBP knockdown in adipocyte cell line 3T3-L1 led to potentiated adipocyte differentiation, upregulation of mitochondrial biogenesis, fatty acid metabolism, peroxisome proliferator-activated receptor γ (PPAR-γ) action, and increased insulin signaling. Cells with LBP knockdown developed resistance to proinflammatory cytokines and other inflammatory stimuli (LPS and palmitate). This phenotype, mediated through disrupted nuclear factor κB (NFκB) signaling, was reversed by exogenous LBP [50]. Congruently, LBP acted as a negative regulator of energy metabolism in adipose tissue in mice and humans [51]. LBP-null mice exhibited the spontaneous induction of subcutaneous adipose tissue browning and increased amount and activity of brown adipose tissue. These changes were associated with decreased weight gain in LBP-null mice and protection against high-fat-diet (HFD)-induced inflammatory responses [51]. Taken together, the present study and previous data suggest a pivotal role of LBP in the pathogenesis of obesity.

### 4.2. BMI Association with Short-Chain Fatty Acids

The present study showed that propionic and butyric, but not acetic or isovaleric acids were significant positive predictors of BMI. These data were in line with previous results showing that serum butyrate [16], as well as fecal propionate [13,14] and butyrate [15], were higher in obese compared to lean individuals, although some studies show no difference in fecal propionic or isovaleric acids [15]. Fecal butyric and propionic acids correlated positively with metabolic risk factors of adiposity, waist circumference and insulin resistance and negatively with HDL [14]. Butyric and propionic acids were decreased in anorexia nervosa compared to normal weight controls [52]. On the other hand, the ex vivo anaerobic incubation of fecal samples from lean and obese individuals with amylose-resistant starch resulted in similar increased production of butyric and propionic acids [53]. Previous studies showing higher levels of SCFAs in obese compared to lean individuals [13,14,15,16] generated the hypothesis that there might be resistance to SCFAs in obesity. This hypothesis was investigated in a few clinical trials of SCFAs supplementation for weight loss and produced divergent results [18,19]. Butyrate supplementation for 45 days did not result in weight loss in a randomized trial of patients with diabetes [18]. In contrast, a novel inulin-propionate ester increased satiety hormones and reduced weight gain compared to the inulin group in a 24-week randomized controlled trial involving 60 overweight adults [19]. Overall, results from the present study and previous research showed multifaceted and divergent roles for SCFAs in weight regulation.

### 4.3. Microbiota Associations with Translocation Markers and SCFAs

The present study suggested some, albeit weak, associations of specific gut microbiota with independent predictors of BMI, including translocation markers LBP and LBP/CD14, as well as propionic acid and cortisol. To the best of our knowledge, there were no other studies evaluating the relationships between permeability markers and microbiota in obese individuals without infectious disorders or inflammatory bowel disease. Specifically, the family Enterobacteriaceae (phylum Proteobacteria) tended to be positively associated with LBP (*p* = 0.03) and CD14 (*p* < 0.01), suggesting a role of Enterobacteriaceae in microbiota-related increased gut permeability that could promote weight gain. The results from the present study were consistent with previous research finding that both fecal and plasma LBP decreased after about 7% weight loss in a dietary intervention trial [54]. The results were also consistent with a study showing that a healthy dietary intake pattern (i.e., higher intake of fruits, yogurt, soups and less sugary drinks) was associated with lower plasma CD14 compared to less healthy dietary intake patterns in a cohort of obese individuals [55]. 

The other bacterial taxa showing possible association with permeability markers was the genus *Catenibacterium* (family Erysipelotrichaceae, phylum Firmicutes). *Catenibacterium* tended to be negatively associated with zonulin (*p* = 0.02), and there was a marginal trend for negative association with BMI (*p* = 0.09), suggesting that lower relative abundance of *Catenibacterium* might be correlated with increased gut permeability and obesity. These data support previous research showing that in vitro fermentation of polysaccharides resulted in the enrichment of *Catenibacterium* in the culture of fecal samples from normal-weight children, while *Enterococcus* was enriched in fecal samples of overweight children [56]. Correspondingly, *Catenibacterium* was enriched in populations eating whole grains and a plant-based vegetarian diet [57,58,59]. In a study on cardiovascular disease risk, *Catenibacterium* and *Alloprevotella* were the only two genera enriched in individuals with low versus high cardiovascular disease risk profiles [32]. These data suggested that, overall, *Catenibacterium* might be related to healthier phenotypes and dietary patterns. The present study also showed that the family Lachnospiraceae (genus *Lachnospira*, phylum Firmicutes) tended to be positively associated with propionic acid (*p* < 0.01), while butyric acid did not reach significance (*p* = 0.10), suggesting possible divergence in bacteria producing these SCFAs. Indeed, the production of propionic and butyric acids by the same bacterium is unusual [60]. Propionate can also be produced by microbiota from phylum Bacteroidetes [61], but we did not detect this association. 

Finally, the genus *Prevotella* (including the species *P. stercorea* and *P. copri*; phylum Bacteroidetes) was also positively associated with cortisol. Although this association was weak, it could represent an important physiological connection. There have been no publications focused on examining the relationship between cortisol and gut microbiota in humans. In goats, chronic dexamethasone exposure increased gut *Prevotella* [62], while in cattle, stress associated with transportation resulted in an initial increase and subsequent decrease of blood cortisol and a similar initial increase and subsequent decline in gut *Prevotella*, suggesting congruent changes in cortisol and *Prevotella* [63]. Previously, *Prevotella* was linked to psychiatric disorders prevalent in our population, including depression [64] and substance use disorder [65], while no studies evaluated association with post-traumatic stress disorder (PTSD). The mechanistic connection of *Prevotella* with cortisol and psychiatric disorders may potentially be explained by the involvement of brain-derived neurotrophic factor (BDNF). Indeed, *Prevotella* was the only genus that had a positive association with plasma BDNF in healthy volunteers [66]. BDNF was also connected to psychiatric disorders prevalent in our population, including depression [67], PTSD [68], and substance use [69]. Moreover, BDNF was shown to have a circadian rhythm that strongly correlated with the cortisol circadian rhythm [37]. These data are suggestive of a cortisol–BDNF–*Prevotella* connection that might be co-regulated and involved in integrated brain activities. 

### 4.4. BMI Association with Cortisol

The current study showed that cortisol was lower in obese compared to non-obese men and was an independent negative predictor of BMI, which is counterintuitive, considering that elevated cortisol is a well-known etiological factor in Cushings’ syndrome. Previous studies showed inconsistent data for the cortisol–obesity relationship. Our data were in accord with previous observations showing a negative association between cortisol and BMI in cross-sectional [70,71,72,73] and longitudinal [70,74] assessment. The other reports, however, showed that the relationship between obesity and cortisol can be positive [75] or U-shaped [71,76]. The recent meta-analysis of 26 studies reporting peripheral cortisol level and BMI showed no significant association between circulating or urinary cortisol and BMI in healthy adults, and also showed that cortisol declined with aging within the obese, but not in the non-obese individuals [77]. In addition, this study population had a high burden of psychiatric conditions known to influence both circulating cortisol and prevalence of obesity [78,79,80,81]. Controlling for psychiatric conditions in our statistical analysis, however, did not change the cortisol–BMI association. One of the multiple possible mechanisms of the obesity–cortisol association could be related to the activity of 11β-hydroxysteroid dehydrogenase type 1 (11β-HSD1) enzyme [82,83,84]. This enzyme encoded by the 11β-HSD1 gene, is highly expressed in abdominal adipose tissue and is responsible for converting inactive cortisone to active cortisol [85]. For example, a longitudinal study of overweight and obese individuals showed that increasing total and trunk fat mass and increased subcutaneous adipose tissue expression of lipogenic genes were associated with decreased expression of subcutaneous adipose tissue 11β-HSD1 gene [84] that could explain, at least in part, lower circulating cortisol in obese individuals. Overall, our data were in agreement with previous research, and suggested that cortisol per se did not lead to obesity (as is the case in Cushings’ syndrome) but appeared to be an important player among multifactorial issues contributing to obesity.

### 4.5. Strengths and Limitations

The study had several strengths and limitations. The strengths of the study were inclusion of population rarely engaged in research and simultaneous inclusion of several microbiota-related biomarkers. Indeed, for the first time, to our knowledge, microbiota metabolic products (SCFAs) and translocation markers (LBP, CD14, EndoCab, zonulin) were included in BMI prediction models along with other relevant variables and showed some of these biomarkers to be significant predictors of BMI in AAM. Limitations included the relatively small group of homogenous participants, as well as the cross-sectional nature of the analysis. Due to the limited resources, we were also unable to capture more detailed lifestyle history and socio-demographic factors, known to contribute to obesity such as stress or sleep disorders. The small sample size prevented accounting for some important possible contributing factors, for example, mental health for microbiota analysis. Finally, many of the associations between microbiota and biomarkers were relatively weak and did not reach significance in FDR-adjusted statistics. Also, only associations and not causations are implied by this analysis.

## 5. Conclusions

The study showed for the first time that gut microbiota biomarkers, including propionic and butyric acids as well as LPS transporters LBP and CD14, were significantly associated with BMI in AAM. Furthermore, our study supported previous research reporting a connection between specific gut microbiota and biomarkers relevant for obesity. The physiological factors included in this study, however, explained only 39%–47% of BMI variability, emphasizing that factors other than microbiota-related biomarkers could be important in the development of obesity. Further research is needed to provide clinical and mechanistic insight into microbiota biomarkers and their utility for diagnostic and therapeutic purposes.

## Figures and Tables

**Table 1:** Characteristics of non-obese and obese men.

Characteristics	Non-Obese (*n* = 34)	Obese (*n* = 41)	*p*-Value
Age, years *	55.3 [5.0]	57.9 [4.0]	0.05
Smoke, *n* [%]	12 [35]	13 [32]	0.94
Exercise, *n* [%] ^a^	15 [44]	23 [56]	0.46
Healthy food/wk *^b^	12.45 [3.50]	10.10 [3.24]	<0.01
Charlson index	1.62 [0.98]	2.15 [0.97]	0.13
Type 2 diabetes, *n* [%] *	1 [3]	37 [90]	<0.01
Psychiatric disorders, *n* [%] *	27 [79]	21 [51]	0.03
Body weight, kg *	79.5 [8.2]	110.4 [11.4]	<0.01
Body mass index, kg/m^2^ *	25.6 [2.4]	35.5 [2.5]	<0.01
HbA1c, % *	5.22 [0.29]	6.70 [0.28]	<0.01
25OHD, ng/mL	17.12 [4.41]	14.86 [4.26]	0.10
LBP, μg/mL *	8.53 [6.70, 10.92]	10.08 [7.19, 15.20]	0.05
EndoCab, GMU/mL	63.67 [53.82, 98.60]	73.39 [49.45, 98.79]	0.65
CD14, ng/mL	1755 [1494, 1903]	1620 [1471, 1761]	0.23
LBP/CD14 × 1000 *	5.10 [4.19, 7.68]	6.45 [5.18, 9.31]	0.01
Zonulin, ng/mL	2.49 [1.47, 6.88]	3.55 [1.96, 5.70]	0.91
Acetic acid, μg/mL	8.46 [5.38, 19.05]	8.90 [7.01, 13.42]	0.89
Propionic acid, μg/mL *	0.92 [0.80, 1.14]	1.72 [1.10, 1.97]	<0.01
Butyric acid, μg/mL *	1.92 [1.63, 2.18]	2.90 [2.23, 3.34]	<0.01
Isovaleric acid, μg/mL *	0.61 [0.56, 0.79]	1.21 [0.80, 1.38]	<0.01
Cortisol, μg/dL *	11.07 [3.32]	9.06 [3.28]	0.02
Testosterone, ng/dL *	401.7 [98.6]	258.8 [105.1]	<0.01

Data are Mean [SD] or Median [interquartile range] for continuous variable and absolute number [%] for categorical variables. All comparisons were adjusted for age. * designates difference *p* ≤ 0.05. ^a^ Exercise was defined as “yes” for 2 h or more per week. ^b^ Healthy food was calculated as combined servings per week of fruits, vegetables, nuts, porridge, breakfast cereal, milk, and tea. Abbreviations: CD14, soluble cluster of differentiation 14 protein; EndoCab, endotoxin core antibody; LBP, lipopolysaccharide-binding protein.

**Table 2:** Associations between body mass index (BMI) and other biomarkers.

Characteristic	Coefficient	*p*-Value
LBP	0.24	0.04
EndoCab	−0.14	0.23
CD14	−0.16	0.17
LBP/CD14	0.31	<0.01
Zonulin	0.12	0.31
Acetic acid	−0.10	0.42
Propionic acid	0.34	<0.01
Butyric acid	0.40	<0.01
Isovaleric acid	0.38	<0.01
Cortisol	−0.33	<0.01
Testosterone	−0.43	<0.01
Healthy food	−0.31	<0.01

Data are partial correlation coefficients, adjusted for age. BMI correlated with age (*r* = 0.28, *p* = 0.01). Abbreviations as in Table 1.

**Table 3:** Models presenting significant determinants for Body mass index (BMI).

	**Butyric Acid and LBP/CD14 (Model-1)**			**Propionic Acid and LBP/CD14 (Model-2)**		
**Characteristic**	**β [SE]**	**95% CI**	***p*-Value**	**β [SE]**	**95% CI**	***p*-Value**
Age	0.05 [0.09]	−0.13, 0.23	0.598	0.09 [0.09]	−0.90, 0.28	0.308
LBP/CD14	3.93 [1.09]	1.74, 6.11	0.001	4.04 [1.14]	1.76, 6.32	0.001
Butyric acid	5.94 [1.72]	2.51, 9.38	0.001			
Propionic acid				3.55 [1.46]	0.64, 6.46	0.018
Testosterone	−1.14 [0.98]	−3.11, 0.82	0.249	−1.23 [1.03]	−3.35, 0.77	0.216
Cortisol	−0.39 [0.13]	−0.66, −0.13	0.005	−0.37 [1.14]	−0.64, −0.09	0.011
Healthy food	−0.44 [0.14]	−0.72, −0.16	0.002	−0.42 [0.15]	−0.71, −0.13	0.006
Psych disorders	−1.54 [1.05]	−3.64, 0.56	0.147	−1.73 [1.09]	−3.92, 0.45	0.118
	**Butyric acid and LBP (Model-3)**			**Propionic acid and LBP (Model-4)**		
**Characteristic**	**β [SE]**	**95% CI**	***p*-Value**	**β [SE]**	**95% CI**	***p*-Value**
Age	0.05 [0.09]	−0.13, 0.24	0.582	1.00 [0.09]	−0.90, 0.29	0.296
LBP	3.33 [1.14]	1.06, 5.60	0.005	3.31 [1.20]	0.92, 5.71	0.007
Butyric acid	6.24 [1.75]	2.74, 9.73	0.001			
Propionic acid				3.78 [1.51]	0.77, 6.79	0.015
Testosterone	−0.98 [1.02]	−3.01, 1.05	0.338	−1.16 [1.07]	−3.29, 0.98	0.282
Cortisol	−0.40 [0.14]	−0.67, −0.13	0.005	−0.37 [1.14]	−0.65, −0.09	0.012
Healthy food	−0.43 [0.14]	−0.72, −0.14	0.004	−0.40 [0.15]	−0.70, −0.11	0.009
Psych disorders	−1.36 [1.08]	−3.52, 0.80	0.214	−1.59 [1.12]	−3.84, 0.66	0.162

Multivariate regression was used for the analysis. Biomarkers with *p* < 0.1 in univariate analysis were used for the final model. The Model-1 and Model-2 explained 47% and 42% of BMI variability when butyric or propionic acid, respectively, were used for modeling. When LBP was used instead of LBP/CD14, the models explain 44% (Model-3) and 39% (Model-4) of BMI variability, respectively. Abbreviations: β, standardized regression coefficient; BMI, body mass index; LBP, lipopolysaccharide-binding protein; Psych, psychiatric.

**Table 4:** Associations of translocation markers and short chain fatty acids with microbiota.

Biomarker	Microbiota (Family; Genus)	τ-Value	*p*-Value	*q*-Value
LBP	Enterobacteriaceae	0.171	0.03	0.58
EndoCab	Alcaligenaceae; *Sutterella*	0.138	0.08	0.99
CD14	Enterobacteriaceae	0.206	<0.01	0.18
	Erysipelotrichaceae; *Catenibacterium*	0.153	0.07	0.64
Zonulin	Erysipelotrichaceae; *Catenibacterium*	−0.188	0.02	0.46
Propionic acid	Lachnospiraceae; *Lachnospira*	0.206	<0.01	0.18
Butyric acid	Lachnospiraceae; *Lachnospira*	0.130	0.10	0.73
	Paraprevotellaceae; *Prevotella*	0.141	0.10	0.73

Data are associations between biomarkers and microbiota. Data are reported for association with *p* ≤ 0.10. Associations were tested between biomarker levels and all taxonomic units using a Kendall Tau (τ) test of correlation. All statistical analyses were performed using R 3.2.3 statistical software. The false-discovery rate (FDR)-adjusted *p*-values (*q*-values) were calculated using the Benjamini–Hochberg FDR correction. Abbreviations: *q*-value, FDR-adjusted *p*-value; τ, correlation coefficient; others as in Table 1.

**Table 5:** Comparisons and correlations of microbiota by cortisol tertiles.

Group Serum Level in T1 vs. T3, Mean [SD]	Cortisol (μg/dL) ^a^ 7.3 [1.7] vs. 16.6 [2.5]	*p*-Value T1 vs. T3	*q*-Value T1 vs. T3	Correlations with Cortisol ^b^ (r-coefficient)	*p*-Value for Correlations with Cortisol
Shannon index	2.35, 2.65	0.006			
**Class**					
Erysipelotrichi	80, 258	<0.01	0.015		
Gammaproteobacteria	78, 218	<0.01	0.08		
Actinobacteria				0.18	0.013
Deltaproteobacteria				0.16	0.033
Bacilli				0.15	0.041
**Order**					
Erysipelotrichales	80, 258	<0.01	0.015		
Enterobacteriales	76, 123	0.04	NS		
Bifidobacteriales				0.16	0.028
Desulfovibrionales				0.16	0.036
**Family**					
Erysipelotrichaceae	80, 258	<0.01	0.015		
Enterobacteriaceae	76, 123	0.04	NS		
Prevotellaceae	284, 523	0.06	NS		
Bacteroidacea	2291, 1492	0.02	NS	−0.15	0.045
Bifidobacteriaceae				0.16	0.028
Desulfovibrionaceae				0.16	0.036
**Genus**					
Veillo; *Phascolarctobacterium*	35, 108	0.07	NS		
Erysipelo; *Catenibacterium*	29, 198	<0.01	0.041		
Prevotella; *Prevotella*	284, 523	0.05	NS		
Bacteroida; *Bacteroides*	2291, 1492	0.02	NS	−0.15	0.045
Lachno; *Lachnospira*				−0.15	0.045
Bifido; *Bifidobacterium*				0.15	0.043
**Species**					
*P. stercorea*	53, 126	0.03	NS		
*P. copri*	219, 382	0.06	NS		

Data are relative abundance of taxa for taxa with *p* < 0.10 in at least one analysis. Shannon index of alpha diversity was assessed by pairwise Mann–Whitney test. ^a^ Data for participants not taking metformin, *p*-values are for the lowest tertile (T1) versus the highest tertile (T3). ^b^ Correlation coefficients r and *p*-values are for cortisol adjusted for BMI in the entire group. Abbreviations: NS, not significant; P, Prevotella; T, Tertiles; vs., versus.
